# Rapid identification of CMV-specific TCRs via reverse TCR cloning system based on bulk TCR repertoire data

**DOI:** 10.3389/fimmu.2022.1021067

**Published:** 2022-11-18

**Authors:** Cheol-Hwa Hong, Hong-Seon Pyo, In-Cheol Baek, Tai-Gyu Kim

**Affiliations:** ^1^ Department of Biomedicine and Health Sciences, College of Medicine, The Catholic University of Korea, Seoul, South Korea; ^2^ Department of Microbiology, College of Medicine, The Catholic University of Korea, Seoul, South Korea; ^3^ Catholic Hematopoietic Stem Cell Bank, College of Medicine, The Catholic University of Korea, Seoul, South Korea

**Keywords:** CMV, TCR - T cell receptor, NGS - next generation sequencing, reverse TCR cloning, artificial APC

## Abstract

Advances in next-generation sequencing (NGS) have improved the resolution of T-cell receptor (TCR) repertoire analysis, and recent single-cell sequencing has made it possible to obtain information about TCR pairs. In our previous study, cytomegalovirus (CMV) pp65-specific T-cell response restricted by a single human leukocyte antigen (HLA) class I allotype was observed in an individual. Therefore, to effectively clone an antigen-specific TCR from these T cells, we developed a TCR cloning system that does not require a single cell level. First, we established the improved Jurkat reporter cell line, which was TCRαβ double knock-out and expressed CD8αβ molecules. Furthermore, functional TCRs were directly obtained by reverse TCR cloning using unique CDR3-specific PCR primers after bulk TCR sequencing of activation marker-positive CD8 T cells by NGS. A total of 15 TCRα and 14 TCRβ strands were successfully amplified by PCR from cDNA of 4-1BB-positive CD8 T cells restricted by HLA-A*02:01, HLA-A*02:06, HLA-B*07:02, and HLA-B*40:06. The panels with combinations of TCRα and TCRβ genes were investigated using Jurkat reporter cell line and artificial antigen-presenting cells (APCs). In two TCR pairs restricted by HLA-A*02:01, one TCR pair by HLA-A*02:06, four TCR pairs by HLA-B*07:02, and one TCR pair by HLA-B*40:06, their specificity and affinity were confirmed. The TCR pair of A*02:01/1-1 showed alloreactivity to HLA-A*02:06. The one TCR pair showed a higher response to the naturally processed antigen than that of the peptide pool. This reverse TCR cloning system will not only provide functional information to TCR repertoire analysis by NGS but also help in the development of TCR-T therapy.

## Introduction

The αβ T-cell receptor (TCR) is a receptor expressed on the surface of αβ T cells, which recognizes antigens presented by major histocompatibility complex (MHC) molecules and triggers a T-cell immune response (1). The human TCR gene has 10^15^–10^21^ potential TCRs through the combination of 47 TRAV, 61 TRAJ, 54 TRBV, 2 TRBD, and 14 TRBJ genes and through the CDR3 generated by random mutations that occur upon recombination (2–4). When T cells are developed in the thymus, they are selected *via* recognition of self- or pathogen-derived antigens presented by self-MHC molecules (5). Through this negative and positive selection, T cells provide specificity for pathogen-derived antigens while preventing autoimmune responses (5, 6). Human leukocyte antigen (HLA), also known as human MHC, is highly polymorphic, with more than 18,000 class I and 7,000 class II allotypes, which leads to many allele-specific peptide binding repertoires that can generally be characterized by sequence motifs (4, 7, 8).

In TCR gene analysis, it was difficult to know the entire sequence of the TCR repertoire with only a pair of primers due to the sequence diversity of the TCR V gene (9). Therefore, a multiplex PCR method using multiple primers specific for various TCR V genes was initially developed, but there was a limitation in that the distribution of TCR repertoire was distorted due to amplification bias and errors (10–13). The subsequently developed method using 5′ RACE and template switching was a simple method that uses a single universal primer, which reduces amplification bias when compared to the multiplex PCR approach but had limitations in template switching efficiency (13–16). Recently, by applying next-generation sequencing (NGS) technology to TCR repertoire analysis, it has become possible to analyze TCR genes at high throughput (13). Moreover, the single-cell technology combined with NGS can provide information on the TCR alpha and beta gene pair to secure a functional TCR gene (13, 15–18). The development of these related analyses and manipulation technologies is accelerating TCR research by overcoming the difficult process of analyzing numerous nucleotide sequences in the TCR repertoire (13, 19).

In the initial studies to obtain a functional antigen-specific TCR, it was necessary to secure T-cell clones by limiting dilution culture (20–22). In subsequent studies, antigen-specific T cells were sorted using epitope peptide-MHC (pMHC) multimers and fluorescence-activated cell sorting (FACS) (18, 23–26). However, epitope-based studies have limitations that cannot be applied to T cells specific for unknown epitopes (18, 25, 26). To overcome this limitation, activation-induced markers (AIMs) such as IFN-γ and 4-1BB have been used to isolate T cells with antigen specificity (18, 24, 27–29). Recent advances in single-cell sequencing technology have made it possible to obtain information about TCR pairs in bulk populations of T cells (18, 30).

Human cytomegalovirus (CMV) can cause acute graft-versus-host disease (GvHD) after allogeneic hematopoietic stem cell transplantation (allo-HSCT) (31, 32). Adoptive transfer of CMV-specific T cells showed restoration of immunological function against CMV infection (33). Antigen-specific TCR for CMV has also been studied to prevent serious disease upon reactivation of CMV (23, 32, 34). In our previous study, to measure T cells specific for pp65, also known as a dominant antigen of CMV, by using artificial antigen-presenting cells (aAPCs) expressing single HLA class I allotypes, it was observed that there was a T-cell response restricted to only one to two single HLA allotypes in an individual (35). If these were proliferated from a small number of T-cell clones, it was expected that antigen-specific TCR could be isolated rapidly with only TCR repertoire data of antigen-specific T cell-enriched population without single cell isolation or T-cell clone culture.

In this study, we could rapidly obtain CMV pp65-specific TCRs by reverse TCR cloning using unique CDR3-specific PCR primers after bulk TCR sequencing of T cells sorted from AIM-positive CD8 T cells. Furthermore, the TCR αβ double knock-out Jurkat cell line, which stably expressed CD8αβ molecules and NFκB signal-based green fluorescent protein (GFP) reporter system, was established and used to confirm the antigen specificity, affinity, and alloreactivity of obtained TCRs.

## Materials and methods

### Peripheral blood mononuclear cells

Four donors were selected (HD18, HD47, HD50, and HD21 as previously described), which were observed to have robust CMV pp65-specific CD8 T-cell responses restricted by HLA-A*02:01, A*02:06, B*07:02, or B*40:06 allotype (35). Primary human peripheral blood mononuclear cells (PBMCs) derived from selected four donors were obtained from the Catholic Hematopoietic Stem Cell Bank. PBMCs were used to expand antigen-specific T cells and analyze the TCR repertoires. Consent forms and approval for this study were obtained from the donors and the Institutional Review Board of The Catholic University of Korea (IRB number: MC17SESI0122).

### Artificial antigen-presenting cell lines

HEK 293T-based aAPCs have been previously described (36) to stably express single HLA class I allotype and co-stimulatory molecules such as CD80, CD83, CD137L, CD54, and CD70. HEK 293T cells (ATCC, cat. no. CRL-3216) and 293T-based aAPCs (HLA null, A*02:01, A*02:06, B*07:02, and B*40:06-293T aAPCs) were cultured in RPMI-1640 medium (Lonza, Durham, NC, USA; cat. no. BE12-702F) supplemented with 10% fetal bovine serum (FBS; HyClone, cat. no. SH30084.03), 1% l-glutamine (Lonza; cat. no. BE17-605E), and 1% penicillin–streptomycin (Lonza; cat. no. DE17-603E). All cells were grown and assayed at 37°C with 5% atmospheric CO_2_.

### Flow cytometry

Target cells were harvested and stained with fluorescent-labeled anti-human antibodies for 30 min at 4°C in the dark, as follows. The following antibodies were used: anti-CD8α-PE (BioLegend, San Diego, CA, USA; cat. no. 300908), anti-CD8α-APC-Cy7 (BioLegend; cat. no. 300926), anti-CD8β-APC (Miltenyi, Bergisch Gladbach, Germany; cat. no. 130-110-569), anti-CD3-BV421 (BioLegend; cat. no. 317344), CMV pp65_495-503_ Tetramer-PE (NLVPMVATV, ProImmune, Oxford, UK), and anti-4-1BB-APC (BioLegend; cat. no. 309810). Fluorescence was measured using a BD FACS Canto or Canto II (BD Biosciences, San Jose, CA, USA) and analyzed using FlowJo software (BD Biosciences).

### Establishment of Jurkat reporter cell line

Jurkat cells (ATCC, Manassas, VA, USA; cat. no. TIB-152) were cultured in RPMI-1640 medium supplemented with 10% FBS, 1% l-glutamine, and 1% penicillin–streptomycin. All cells were grown and assayed at 37°C with 5% atmospheric CO_2_. Lentiviral transduction was used to establish a Jurkat-based reporter cell line by HLA class I-mediated TCR signaling. For the production of lentivirus, 5 × 10^6^ 293T cells/10 ml were seeded in T75 flasks. After 24 h, 10 μg of a pCDH plasmid (SBI, Palo Alto, CA, USA; cat. no. CD523A-1) encoding CD8β-T2A-CD8α or 10 μg of a pGF1-NFκB plasmid (SBI; cat. no. TR012PA-1) was co-transfected into 293T cells with lentivirus packaging plasmids (5 μg of pMD2.G and 5 μg of psPAX2; Addgene, Watertown, MA, USA; cat. nos. 12259, 12260) using Lipofectamine 2000 reagent (Invitrogen, Carlsbad, CA, USA; cat. no. 11668-019). At 48 h after transfection, lentiviral supernatants were harvested and filtered through 0.45-μm filters. To stably express CD8αβ molecules and NFκB transcriptional reporter system *via* transduction of each lentivirus, 5 × 10^5^ Jurkat cells/ml were seeded on 6-well plates. After 24 h, 500 μl of lentiviral supernatant and 8 μg/ml of polybrene were added to Jurkat cell cultures. At 48 h after transduction, cells were cultured and analyzed by flow cytometry. Jurkat cells were transduced with lentiviruses encoding CD8β-T2A-CD8α and NFκB transcriptional reporter system stimulated with 2 μl of Dynabeads (Gibco, Grand Island, NY, USA; cat. no. 11161D) for 24 h. Stimulated cells were harvested in autoMACS Rinsing Solution (Miltenyi; cat. no. 130-091-222) and stained with anti-CD8β-APC for 30 min at room temperature. Live, CD8β-positive, GFP-positive Jurkat cells were sorted, and single cells were seeded in 96-well plates using Moflo XDP Cell Sorter (Beckman, Brea, CA, USA). Finally, single cells expressing high levels of CD8αβ and GFP after stimulation with 2 μl of Dynabeads were cloned and selected (J8G clone).

### Endogenous T-cell receptor knock-out in Jurkat reporter cell line

To eliminate the expression of endogenous TCR, two gRNAs were designed, targeting *TRAC* (GACACCTTCTTCCCCAGCCC) and *TRBC* (CCACGTGGAGCTGAGCTGGT). All-in-one plasmids including both gRNA and Cas9 gene were obtained from GenScript (Cat. no. PX458). For the establishment of the TCRβ knock-out J8G clone, the J8G clone was transfected with *TRBC* target CRISPR/Cas9 plasmid. J8G cells were centrifuged at 1,800 rpm for 3 min, and cell pellets were resuspended in Opti-MEM (Gibco; cat. no. 31985070). *TRBC* target CRISPR/Cas9 plasmid measuring 10 μg was added to 1 × 10^6^ J8G cells resuspended in 200 μl of Opti-MEM. The electroporation protocol was 400 V with a 500-μs pulse (ECM 830, BTX, Holliston, MA, USA). At 9 days after transfection, cells were harvested in autoMACS Rinsing Solution and stained with anti-CD3-BV421 for 30 min at room temperature. Live, CD3-negative J8G cells were sorted, and single cells were seeded in 96-well plates using Moflo XDP Cell Sorter. For the establishment of the TCRαβ double knock-out J8G clone, the TCRβ knock-out J8G clone (J8GB) was transfected with *TRAC* target CRISPR/Cas9 plasmid. J8GB cells were centrifuged at 1,800 rpm for 3 min, and cell pellets were resuspended in Opti-MEM. *TRAC* target CRISPR/Cas9 plasmid measuring 10 μg was added to 1 × 10^6^ J8GB cells resuspended in 200 μl of Opti-MEM. The electroporation protocol was 400 V with a 500-μs pulse (ECM 830, BTX). At 12 days after transfection, cells were transfected with 30 μg of TCRβ IVT mRNA using BTX electroporation protocol (400 V with a 500-μs pulse). At 24 h after transfection, cells were harvested in autoMACS Rinsing Solution and stained with anti-CD3-BV421 for 30 min at room temperature. Live, CD3-negative J8GB cells were sorted, and single cells were seeded in 96-well plates using Moflo XDP Cell Sorter. At 2–3 weeks after sorting, single-cell clones were established. To select the final cell line, TCRαβ double knock-out J8G clones were transfected with IVT mRNAs of a previously reported LMP1-specific TCR (37) and stimulated with 10 μM of LMP1_166-174_ peptide (TLLVDLLWL) pulsed on aAPCs expressing HLA-A*02:01 allotype for 24 h. Among the TCRαβ double knock-out J8G clones, the 3G2 clone (J8GAB) was finally selected, which has the highest GFP expression after antigen-specific stimulation, and the cDNA nucleotide sequences of TCRα and TCRβ were analyzed by Sanger sequencing (Cosmo Genetech, Seoul, Korea).

### Expansion and isolation of cytomegalovirus pp65-specific T cells

To expand antigen-specific T cells, PBMCs were adjusted to 3 × 10^6^ cells/2 ml and cultured in complete RPMI-1640 media supplemented with 1,000 U of IFN-γ (PeproTech, Cranbury, NJ, USA; cat. no. AF-300-02) and 1 μg of CMV pp65 peptide pool (JPT, Berlin, Germany; cat. no. PM-PP65-2). On day 1, 600 U of IL-2 (Proleukin) was added to each well. PBMC density was adjusted to 1 × 10^6^ cells/2 ml on days 4, 7, and 11, and the cells were re-stimulated with 300 U/ml of IL-2 and 5 ng/ml of IL-15 (PeproTech; cat. no. AF-200-15). On day 13, stimulated PBMCs were harvested and stimulated with 10 μM of CMV pp65 peptide pool pulsed on each matched aAPCs expressing a single HLA class I allotype for 24 h (APC : PBMC ratio; 1:10). Stimulated PBMCs captured IFN-γ-secreting cells by using IFN-γ Secretion Assay Detection Kits (Miltenyi; cat. no. 130-054-202) according to the manufacturer’s instructions and stained with anti-CD3-BV421, anti-CD8α-APC-Cy7, and anti-4-1BB-APC for 30 min at 4°C. In another test tube, stimulated PBMCs were stained with CMV pp65_495-503_ Tetramer-PE, anti-CD3-BV421, and anti-CD8α-APC-Cy7 for 30 min at 4°C. In CD3-positive and CD8-positive cells, tetramer, IFN-γ, or 4-1BB-positive cells were sorted using SH800S Cell Sorter (Sony, San Jose, CA, USA). Sorted cells were used for the analysis of TCR repertoires by TCR sequencing.

### The T-cell receptor sequencing by next-generation sequencing

With the use of sorted CMV pp65-specific T cells, the TCR repertoire libraries were prepared by the SMARTer Human TCR a/b Profiling Kit (Takara, Mountain View, CA, USA; cat. no. 635015) according to the manufacturer’s instructions. TCR repertoire libraries were sequenced (Macrogen, Seoul, Korea) by Illumina MiSeq (Illumina, San Diego, CA, USA) with MiSeq Reagent Kit v3 (Illumina; cat. no. MS-102-3003). Data were analyzed by MiXCR and VDJtools, which enable the profiling and measurement of clonotypes, statistical analysis, and visualization of the results.

### Reverse T-cell receptor cloning based on T-cell receptor repertoire data

Through analysis using MiXCR, clonotypes occupying more than 4.5% of the total proportion in 4-1BB-positive sorted CD8 T cells were selected as candidates for functional TCRα and TCRβ pairs. Based on V gene usages and CDR3 nucleotide sequences of selected TCRs, primers were designed to amplify only target TCR, which had specific CDR3 and V usage from the TCR gene pool. Primer sequences are provided in **Table S2**. The PCR was performed using KOD FX (Toyobo, Osaka, Japan; cat. no. KFX-101). To amplify the total TCR gene pool, intermediate products of TCR repertoire libraries were used as a template, and PCR was performed with forward primer including sequence universal switch oligo of libraries and TCR constant reverse primers. The PCR program was as follows: 94°C for 2 min, 35 cycles of 98°C for 10 s, 60°C for 30 s, 68°C for 90 s, and extension at 68°C for 5 min. The PCR products were column purified by using the NucleoSpin Gel and PCR Clean-up kit (Macherey-Nagel, Düren, Germany; cat. no. MN740609.250). The purified PCR products were used as templates for CDR3-targeted PCR. CDR3-targeted PCR was performed using two pairs of primers to target the CDR3 sequence, each targeting CDR3 in the variable region (including part of the T7 promoter sequence) and CDR3 to the constant region (containing part of the poly A signal sequence). The PCR program was as follows: 94°C for 2 min, 35 cycles of 98°C for 10 s, 60°C for 30 s, 68°C for 45 s, and extension at 68°C for 5 min. To obtain a complete IVT template of TCRs, overlapping PCR was performed with two products of CDR3-targeted PCR as templates and complete T7 promoter forward primer and beta-globin poly(A) signal reverse primer. The PCR program was as follows: 94°C for 2 min, 35 cycles of 98°C for 10 s, 60°C for 30 s, 68°C for 90 s, and extension at 68°C for 5 min. The PCR products were column purified by using the NucleoSpin Gel and PCR Clean-up kit. The purified PCR products (300 ng/μl) were used as templates for IVT. The IVT was performed using MEGAscript T7 Transcription Kit (Invitrogen; cat. no. AM1334) according to the manufacturer’s instructions with ARCA (TriLink, San Diego, CA, USA; cat. no. N-7003-10) performed overnight, and polyadenylation was performed using Poly(A) Tailing Kit (Invitrogen; cat. no. AM1350). Final IVT products were purified by using the MEGAclear Transcription Clean-Up Kit (Invitrogen; cat. no. AM1908) according to the manufacturer’s instructions. To identify pairs of CMV pp65-specific TCR from selected TCRs, combinations of TCRα and TCRβ IVT mRNAs (30 μg of each chain) were transfected into the J8GAB cells using BTX electroporation protocol (400 V with a 500-μs pulse). To stimulate HLA-restricted antigen, aAPCs expressing each matched HLA class I allotype were pulsed with 1 μM of CMV pp65 peptide pool for 4 h. TCR-transfected J8GAB cells (1 × 10^5^) co-cultured with aAPCs (1 × 10^4^) in a volume of 200 μl of complete RPMI-1640 media in flat-bottom 96-well plates for 18 h. Cells were centrifuged, and cell pellets were resuspended in PBS (Lonza; cat. no. BE17-516F) supplemented with 2% FBS. J8GAB cells that transferred TCRs were stained with anti-CD3-BV421 to analyze the expression of transferred TCRs. J8GAB cells that transferred TCRs and co-cultured with aAPCs were measured to analyze the expression of GFP and the function of transferred TCRs.

### Determination of affinity, epitope, and alloreactivity of cytomegalovirus pp65-specific T-cell receptors

To determine the affinity of selected TCRs, aAPCs expressing HLA-A*02:01, HLA-A*02:06, HLA-B*07:02, or HLA-40:06 were pulsed with a 10-fold dilution pp65 peptide pool (from 10^1^ to 10^−6^ μM) for 4 h; 1 × 10^5^ J8GAB cells expressing pp65 reactive TCRs were co-cultured with each matched 1 × 10^4^ aAPC in a volume of 200 μl of complete RPMI-1640 media in flat-bottom 96-well plates for 18 h. Cells were measured for the expression of GFP induced by each pp65 concentration and were converted to a stimulus–response curve through normalization. Half maximal effective concentration (EC50) was calculated by GraphPad Prism 9 (GraphPad). To determine the epitope of selected TCRs, candidates of epitope restricted by HLA allotypes were selected based on the Immune Epitope Database (IEDB). HLA-A*02:01 and HLA-A*02:06 epitope peptides (NLVPMVATV; JPT; cat. no. SP-MHCI-0005) and HLA-B*07:02 epitope peptides (RPHERNGFTVL and TPRVTGGGAM; JPT; cat. no. SP-MHCI-0013 and SP-MHCI-0014) were pulsed on each matched aAPC expressing a single HLA allotype for 4 h (10 μM). IVT mRNA expressing HLA-B*40:06 epitope (AELEGVWQPA) measuring 10 μM was transfected into aAPCs expressing a single HLA-B*40:06 allotype. aAPCs pulsing or expressing epitope measuring 1 × 10^4^ were co-cultured with 1 × 10^5^ J8GAB cells expressing pp65 reactive TCRs in a volume of 200 μl of complete RPMI-1640 media in flat-bottom 96-well plates for 18 h. The expression of GFP induced by epitope stimulation was measured by flow cytometry. In the investigation of alloreactivity of selected TCRs, 18 types of pCDH plasmid encoding each single HLA class I allotype (HLA-A*02:01, HLA-A*02:06, HLA-A*11:01, HLA-A*24:02, HLA-A*30:01, HLA-A*31:01, HLA-A*33:03, HLA-B*07:02, HLA-B*13:02, HLA-B*15:01, HLA-B*35:01, HLA-B*40:01, HLA-B*40:06, HLA-B*44:03, HLA-B*48:01, HLA-B*51:01, HLA-B*52:01, and HLA-C*03:04) were used; 5 μg of a pCDH plasmid encoding a single HLA class I allotype was transfected into HLA-null aAPCs. Twenty-four hours after transfection, 1 × 10^5^ J8GAB cells expressing pp65 reactive TCRs were co-cultured with 1 × 10^4^ aAPCs expressing single HLA class I allotype by transfection in a volume of 200 μl of complete RPMI-1640 media in flat-bottom 96-well plates for 18 h. The expression of GFP induced by each HLA class I allotype was measured by flow cytometry.

### Determination of reactivity of cytomegalovirus pp65-specific T-cell receptors to naturally processed antigen

CMV pp65-T2A-TagBFP template of IVT reaction was generated by nested PCR with T7 promoter forward primer and beta-globin poly(A) signal reverse primer. Primer sequences are given in **Table S2**. First, PCR was target specific to add sequences including part of the T7 promoter and beta-globin poly(A) signal to 5′ and 3′ ends. The PCR program was as follows: 94°C for 2 min, 35 cycles of 98°C for 10 s, 60°C for 30 s, 68°C for 4 min, and extension at 68°C for 5 min. The PCR products were gel purified by using the NucleoSpin Gel and PCR Clean-up Kit. The purified products were used as templates for the second PCR. Second, PCR was performed with complete T7 promoter forward primer and beta-globin poly(A) signal reverse primer. The PCR program was as follows: 94°C for 2 min, 35 cycles of 98°C for 10 s, 60°C for 30 s, 68°C for 4 min, and extension at 68°C for 5 min. The PCR products were column purified by using the NucleoSpin Gel and PCR Clean-up kit. The purified PCR products (300 ng/μl) were used as templates for IVT. The IVT was performed using the MEGAscript T7 Transcription Kit according to the manufacturer’s instructions with ARCA performed overnight, and polyadenylation was performed using the Poly(A) Tailing Kit. Final IVT products were purified by using the MEGAclear Transcription Clean-Up Kit according to the manufacturer’s instructions. CMV pp65-T2A-TagBFP IVT mRNA measuring 1 μM was transfected into the aAPCs using BTX electroporation protocol (400 V with a 500-μs pulse); 1 × 10^5^ J8GAB cells expressing pp65 reactive TCRs were co-cultured with 1 × 10^4^ aAPCs expressing a single HLA class I allotype and CMV pp65-T2A-TagBFP by transfection in a volume of 200 μl of complete RPMI-1640 media in flat-bottom 96-well plates for 18 h. The expression of GFP induced by antigen-specific stimulation *via* naturally processed CMV pp65 was measured by flow cytometry.

## Results

### Establishment of Jurkat reporter cell line to identify T-cell receptor

To effectively analyze the characteristics of transferred TCR genes, we established a Jurkat cell-based reporter cell line in which GFP expression was induced by NFκB signal, both CD8α and CD8β molecules were stably expressed, and the endogenous TCR expression was eliminated.

**Figure 1:**
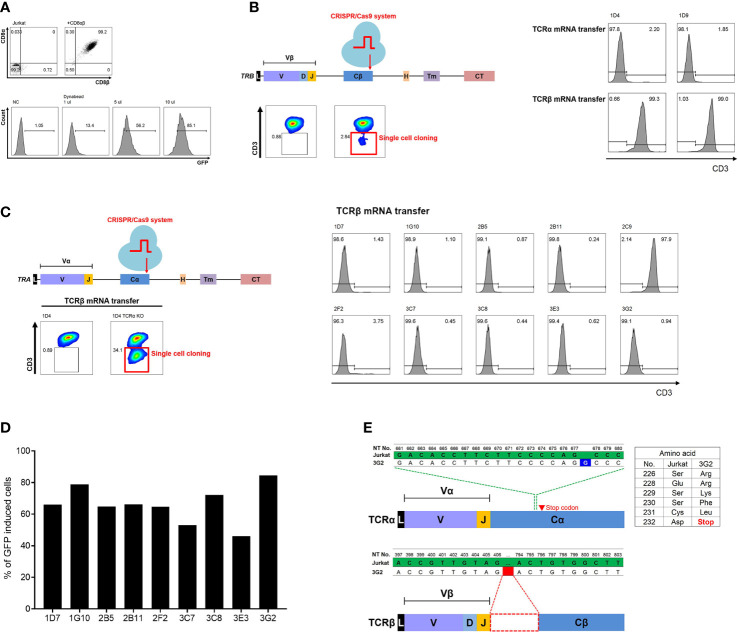
Establishment of TCRαβ double knock-out Jurkat cell line using CRISPR/Cas9 system to identify TCR. **(A)** Phenotypic analysis of Jurkat cell line (J8G clone) expressing CD8αβ and inducing GFP *via* NFκB signaling. Expression of CD8αβ and induced GFP *via* NFκB signaling were analyzed by flow cytometry. When J8G clones were stimulated with beads coated with anti-CD3 and anti-CD28 antibodies, it was observed that the expression of GFP increased with stimulation intensity. **(B)** *TRBC* gene knock-out using CRISPR/Cas9 system in J8G clone. To confirm TCRβ knock-out in clones not expressing CD3 after TCRβ knock-out, the recovery of CD3 expression after transfection of LMP1-specific TCRα or TCRβ mRNA was measured. In 1D4 and 1D9 clones among nine clones not expressing CD3, TCRβ mRNA transfection showed recovery of CD3 expression, but CD3 expression did not restore despite TCRα mRNA transfection. Therefore, 1D4 and 1D9 clones were finally selected as TCRβ knock-out clones. **(C)** Additional *TRAC* gene knock-out using CRISPR/Cas9 system in *TRBC* gene knock-out 1D4 clone (J8GB). To determine TCRαβ double knock-out clones, 10 clones were obtained by sorting cells that still did not express CD3 despite TCRβ mRNA delivery after *TRAC* gene knock-out using the CRISPR/Cas9 system. Nine clones did not restore CD3 expression despite repeated TCRβ mRNA transfections. These nine clones were selected as TCRαβ double knock-out clones. **(D)** Analysis of GFP induction *via* antigen-specific stimulation in TCRαβ double knock-out clones. TCRαβ double knock-out clones were transfected with LMP1-specific TCRα and TCRβ mRNA and stimulated with LMP1 epitope peptide pulsed at single HLA-A*02:01-expressing aAPCs. The 3G2 clone showed the highest GFP induction and was selected as a clone (J8GAB) for the identification of functional TCRs. **(E)** Analysis of cDNA sequences in J8GAB. In order to confirm the transcribed TCRα and TCRβ sequences, mRNA was isolated from the J8GAB. The TCRα sequence had a single guanine inserted (blue) between nucleotides 677 and 678. This single guanine insertion resulted in frameshifting and premature termination of translation. The TCRβ sequence had a large deletion (red) from nucleotides 407 to 793. TCR, T-cell receptor; GFP, green fluorescent protein; aAPCs, artificial antigen-presenting cells.

First, in order to effectively recognize the antigen presented by HLA class I, CD8α and CD8β molecules, which are co-receptors of TCR of CD8 T cells, were transduced into a CD4-positive Jurkat cell line to ensure stable expression. As a next step, we established the Jurkat cell line in which GFP expression is induced by NFκB signaling through TCR to identify the function of transferred TCR by flow cytometry. Finally, one Jurkat clone (J8G) with the highest expression of CD8αβ molecules and efficient GFP induction was selected. The J8G clone showed 99.2% CD8α and CD8β expression (**Figure 1A**). After 24 h of stimulation with 1, 5, and 10 μl of anti-CD3 and anti-CD28 antibodies-coated beads in 10^5^ cells, the J8G clone showed 13.4%, 56.2%, and 85.1% GFP-positive cells, respectively (**Figure 1A**).

In order to accurately measure the specificity of the transferred TCR and increase cell surface expression, it was necessary to completely eliminate the endogenous TCR of the J8G clone. To eliminate the endogenous TCRα and TCRβ using the CRISPR/Cas9 system, the constant region of TCR was selected as a target of TCR knock-out. Since the surface expression of CD3 is linked according to the expression of the complete TCR pair, CD3 expression was measured as a marker for TCR gene knock-out. After *TRBC* gene target CRISPR/Cas9 transfection, CD3-negative J8G cells were 2.84% and cultured as single-cell clones (**Figure 1B**). To confirm the TCRβ knock-out, LMP1-specific TCRα or TCRβ mRNA was transfected into nine CD3-negative clones. CD3 expression in J8G-1D4 and J8G-1D9 clones was 2.2% and 1.85% after TCRα mRNA transfection and 99.3% and 99% after TCRβ mRNA transfection, respectively (**Figure 1B**). In addition, for complete clearance of the endogenous TCR, we tried to knock out TCRα in the J8G-1D4 clone (J8GB). At 13 days after *TRAC* gene target CRISPR/Cas9 transfection, CD3-negative cells were 34.1% despite TCRβ mRNA transfection, and these cells were cultured as single-cell clones (**Figure 1C**). CD3 expression in nine clones except the 2C9 clone was less than 4% after TCRβ mRNA transfection (**Figure 1C**).

We compared the intensity of GFP induction in nine TCRαβ double knock-out clones to select the final clone to be used to identify functional TCR. These clones were transfected with LMP1-specific TCR mRNA and stimulated with LMP1_166-174_ peptide (TLLVDLLWL) pulsed on aAPCs expressing a single HLA-A*02:01. Among the nine clones, the J8G-1D4-3G2 clone showed the highest GFP induction and 92.1% of GFP-positive cells after antigen-specific stimulation (**Figure 1D**, **Supplementary Figure S1**). In Sanger sequencing data of the TCR cDNA from the J8G-1D4-3G2 clone, the TCRα sequence had a single guanine insertion between nucleotides 677 and 678, which resulted in frameshifting and premature termination of translation (**Figure 1E**). The TCRβ sequence had a large deletion from nucleotides 407 to 793 (**Figure 1E**). Through flow cytometry and cDNA sequencing, the J8G-1D4-3G2 clone was finally selected for the identification of functional TCRs and named J8GAB cells in subsequent experiments.

### Comparison of T-cell receptor repertoire according to sorting markers

**Figure 2:**
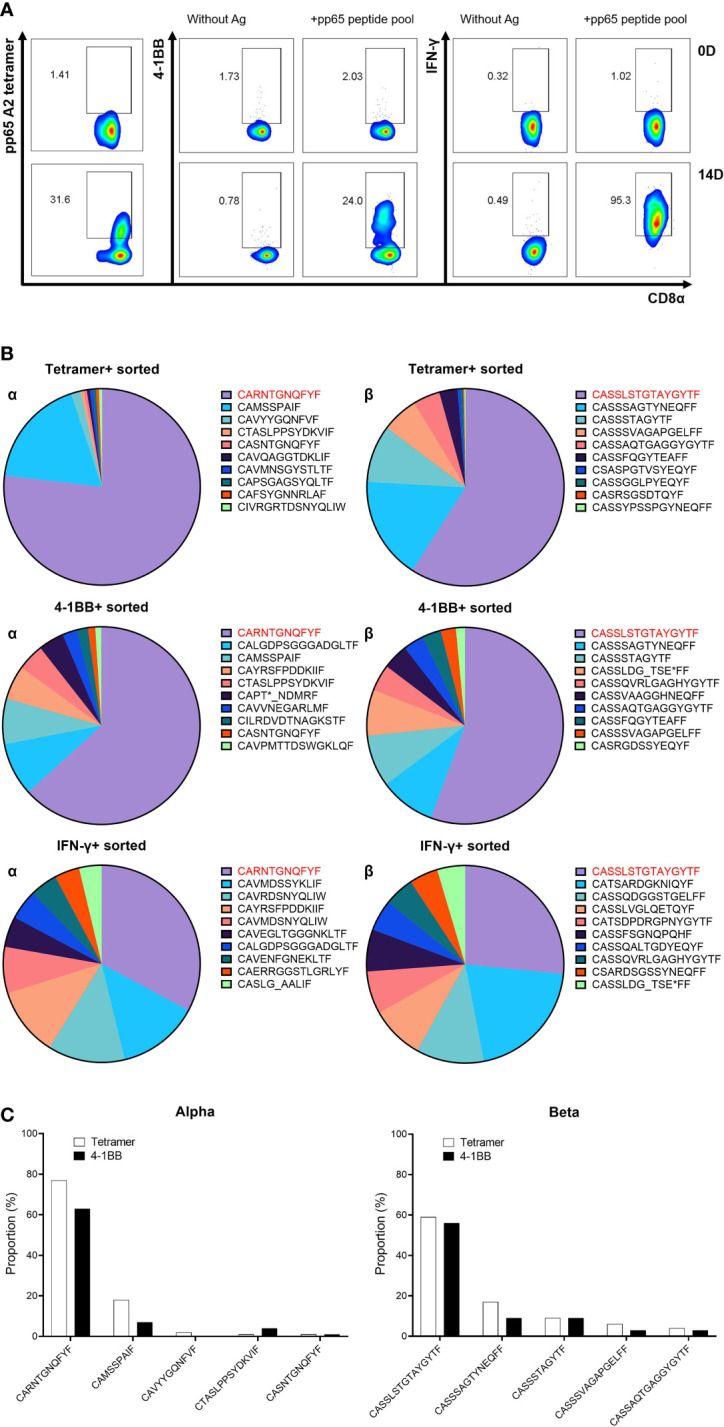
Comparison of TCR repertoire according to methods for isolating antigen-specific T cells. **(A)** Flow cytometric analysis of three sorting markers—pp65 tetramer, 4-1BB, and IFN-γ—on 14D cultured CD8 T cells. PBMCs isolated from selected donors (HD18) with robust T-cell responses to the CMV pp65 antigen were stimulated with the pp65 peptide pool. At 13 days after stimulation, CD8 T cells were re-stimulated with pp65 peptide pool pulsed on aAPCs expressing HLA-A*02:01 for 24 h, and then pp65 tetramer-, 4-1BB-, and IFN-γ-positive CD8 T cells were analyzed. **(B)** TCR repertoire analysis by NGS on tetramer or activation-induced marker (AIM)-positive sorted CD8 T cells. mRNAs were isolated from each sorted CD8 T cells, and libraries synthesized using TCR sequencing kit were analyzed with NGS. Top 10 ranked CDR3 sequences were compared between pp65 tetramer-, 4-1BB-, and IFN-γ-positive CD8 T cells (red: top CDR3 sequence). **(C)** Direct comparison of CDR3 sequences between pp65 tetramer- and 4-1BB-positive CD8 T cells. Comparing the high proportion of CDR3 sequences, these two markers showed high similarity. In order not to depend on peptides, 4-1BB was used as an antigen-specific T-cell sorting marker in future studies. TCR, T-cell receptor; PBMCs, peripheral blood mononuclear cells; aAPCs, artificial antigen-presenting cells; NGS, next-generation sequencing.

Analysis of TCR repertoire was performed to select a proper marker for sorting antigen-specific CD8 T cells. Based on data from a previous study, an HD18 donor with strong CMV pp65-specific T-cell responses restricted by HLA-A*02:01 was used in this study (35). CMV pp65-specific CD8 T cells from PBMCs of HD18 were cultured with the pp65 peptide pool. After 13 days of culture, the CD8 T cells were re-stimulated by aAPCs expressing HLA-A*02:01 with the pp65 peptide pool for 24 h. A portion of CMV pp65-specific CD8 T cells was detected by flow cytometry analysis on three markers: pp65 A2 tetramer staining, surface 4-1BB expression, and IFN-γ capture assay. Stimulated CD8 T cells showed 38.8%, 24%, and 95.3% of pp65 A2 tetramer-, 4-1BB-, and IFN-γ-positive cells, respectively (**Figure 2A**). It was confirmed that the CMV pp65-specific CD8 T cells that were positive in the three markers after culture were more increased than those of the CD8 T cells before culture (**Figure 2A**). The TCR repertoire of CD8 T cells sorted according to each marker was analyzed, and the top 10 TCR repertoire data were compared (**Figure 2B**). The most frequent amino acid sequences of the CDR3α and CDR3β were all identical, and those in the lower ranks had some identical ones, but the order was different (**Figure 2B**). In particular, the distribution in the 4-1BB group was most similar to that in the tetramer group (**Figure 2C**). Therefore, 4-1BB was used as a marker to isolate antigen-specific T cells in subsequent experiments.

### Reverse T-cell receptor cloning based on T-cell receptor repertoire data

**Figure 3:**
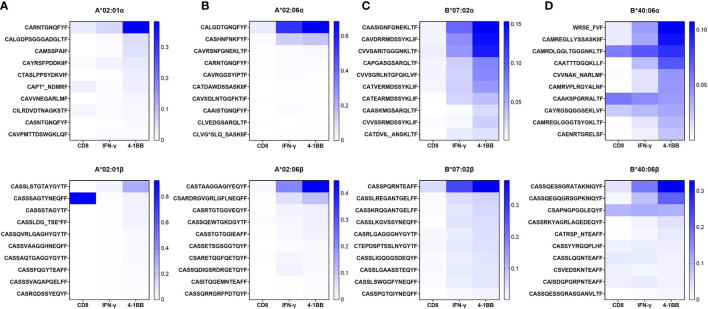
TCR repertoire analysis of CMV pp65-specific CD8 T cells restricted by single HLA class I allotype. In previous study, HD18, HD47, HD50, and HD21, the four selected donors, were found to have robust CMV pp65-specific CD8 T-cell responses restricted by HLA-A*02:01, A*02:06, B*07:02, or B*40:06 allotype, respectively. PBMCs were stimulated with the pp65 peptide pool for 13 days and then were re-stimulated for 24 h with the pp65 peptide pool pulsed on aAPCs expressing single HLA class I allotype. TCR repertoire of sorted total CD8 T cells and 4-1BB- and IFN-γ-positive CD8 T cells were analyzed by NGS. The top 10 ranked CDR3 sequences for each sorted group were displayed as heatmaps. **(A)** Proportion of CDR3α and CDR3β sequences of CD8 T cells restricted by HLA-A*02:01. **(B)** Proportion of CDR3α and CDR3β sequences of CD8 T cells restricted by HLA-A*02:06. **(C)** Proportion of CDR3α and CDR3β sequences of CD8 T cells restricted by HLA-B*07:02. **(D)** Proportion of CDR3α and CDR3β sequences of CD8 T cells restricted by HLA-B*40:06. TCR, T-cell receptor; CMV, cytomegalovirus; PBMCs, peripheral blood mononuclear cells; aAPCs, artificial antigen-presenting cells; NGS, next-generation sequencing; HLA, human leukocyte antigen.

CMV pp65-specific T cells were cultured in four donors (HD18, HD47, HD50, and HD21) with strong T-cell responses restricted by HLA-A*02:01, HLA-A*02:06, HLA-B*07:02, or HLA-B*40:06 and isolated in the same method after 14 days of culture. TCR repertoire was analyzed in total CD8 T cells, 4-1BB-positive CD8 T cells, and IFN-γ-secreting CD8 T cells. Based on the TCR repertoire data of 4-1BB-positive CD8 T cells, 10 CDR3α and CDR3β sequences showing high frequency were listed in order and compared with the data of total CD8 T cells or IFN-γ-secreting CD8 T cells (**Figure 3**). In 4-1BB-positive CD8 T cells, higher-frequency CDR3 sequences were more frequent than total CD8 T cells or IFN-γ-secreting CD8 T cells. These results suggest that the existence of antigen-specific T-cell clones can be easily observed in isolated CD8 T cells after antigen stimulation using aAPC with a single HLA allotype.

**Figure 4:**
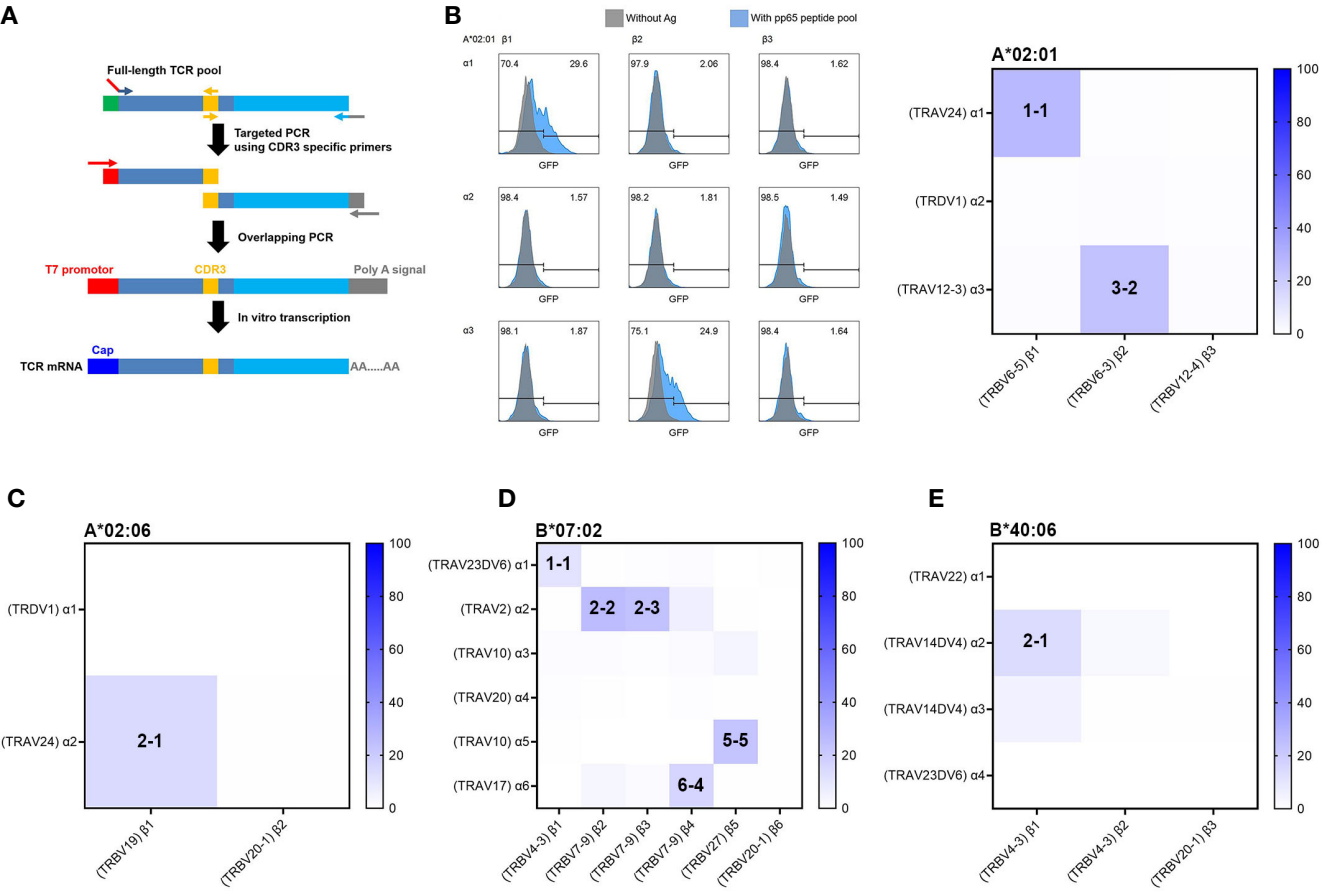
Reverse TCR cloning and identification of functional TCR using aAPCs and Jurkat reporter cell line. **(A)** Schematic summary of reverse TCR cloning from cDNAs of sorted T cells based on candidate TCR sequences. Based on the TCR sequences of 4-1BB-sorted CD8 T cells, TCR sequences occupying more than 4.5% of the total proportion were selected as candidates for functional TCRα and TCRβ pairs. Full-length TCR pools were amplified from cDNAs of 4-1BB-positive sorted CD8 T cells by first PCR. For CDR3-targeted PCR, second PCR was performed using two pairs of primers to target the CDR3 sequence, each targeting CDR3 in the variable region (including part of the T7 promoter sequence) and CDR3 to the constant region (containing part of the poly A signal sequence). A third overlapping PCR was performed with second PCR products using the T7 promoter forward primer (red) and the poly **(A)** tailing signal reverse primer (gray). The product of the third overlapping PCR was used as a template for IVT. Combinations of TCRα and TCRβ IVT mRNAs were transfected into the J8GAB cells for determination of antigen-specific reactivity. **(B)** Representative flow cytometry and heatmap results for GFP expression after antigen-specific stimulation of the J8GAB cells. Combinations of TCRα and TCRβ IVT mRNAs were transfected into the J8GAB cells for determination of antigen-specific reactivity. J8GAB cells expressing TCR were stimulated with the pp65 peptide pool pulsed on aAPCs expressing HLA-A*02:01. The TCR pairs of α1-β1 (A*02:01/1-1) and α3-β2 (A*02:01/3-2) showed GFP induction. **(C)** Heatmap results for GFP expression after stimulation of the J8GAB cells expressing TCR with the pp65 peptide pool pulsed on aAPCs expressing HLA-A*02:06. The TCR pairs of α2-β1 (A*02:06/2-1) showed GFP induction. **(D)** Heatmap results for GFP expression after stimulation of the J8GAB cells expressing TCR with the pp65 peptide pool pulsed on aAPCs expressing HLA-B*07:02. The TCR pairs of α1-β1 (B*07:02/1-1), α2-β2 (B*07:02/2-2), α2-β3 (B*07:02/2-3), α5-β5 (B*07:02/5-5), and α6-β4 (B*07:02/6-4) showed GFP induction. **(E)** Heatmap results for GFP expression after stimulation of the J8GAB cells expressing TCR with the pp65 peptide pool pulsed on aAPCs expressing HLA-B*40:06. The TCR pairs of α2-β1 (B*40:06/2-1) showed GFP induction. TCR, T-cell receptor; aAPCs, artificial antigen-presenting cells; GFP, green fluorescent protein.

Typically, to obtain functional TCR pairs, TCRs have been cloned from T-cell clones or, more recently, from single T cells. As in this study, TCR repertoire analysis data by bulk TCR sequencing for simply isolated antigen-specific T cells are mixed with TCR cDNA sequences of various clones and do not provide information on TCR pairs but can provide genetic information of individual TCR clones. Therefore, we developed a reverse TCR cloning method that uses TCR repertoire data to directly obtain the cDNA of candidate antigen-specific TCR with high frequency through PCR amplification specific for the unique CDR3 sequence of each T-cell clone (**Figure 4A**). Briefly describing the process, first, the PCR product of the variable region was generated using a CDR3 sequence-specific reverse primer and a forward primer containing a start codon, and the PCR product of the constant region was also generated using a CDR3 sequence-specific forward primer and a reverse primer containing a stop codon. In the following step, overlapping PCR was performed using the forward primer containing the T7 promoter and the reverse primer containing the poly(A) signal to finally obtain the complete TCR gene. A total of 15 TCRα and 14 TCRβ strands were successfully amplified by PCR using the cDNA of 4-1BB-positive CD8 T cells. From the cDNA of 4-1BB-positive CD8 T cells restricted by HLA-A*02:01, three TCRα and three TCRβ strands were obtained. From the cDNA of 4-1BB-positive CD8 T cells restricted by HLA-A*02:06, two TCRα and two TCRβ strands were obtained. From the cDNA of 4-1BB-positive CD8 T cells restricted by HLA-B*07:02, six TCRα and six TCRβ strands were obtained. From the cDNA of 4-1BB-positive CD8 T cells restricted by HLA-B*40:06, four TCRα and three TCRβ strands were obtained. Detailed information on each TCR is provided in **Table S1**.

### Identification of functional T-cell receptors by combination of candidate T-cell receptor pairs

Since TCRα and TCRβ cDNAs were obtained independently, a combination of TCRα and TCRβ *in vitro* transcription (IVT) mRNA was transfected into the J8GAB cells in order to determine functional TCR pairs with antigen specificity. When TCRα and TCRβ IVT mRNAs were co-transfected, the efficiency was higher than an average of 84.3% (**Supplementary Figure S2**). CMV pp65-specific reactivity of the TCR pair was confirmed by GFP induction of J8GAB cells after stimulation with pp65 peptide pool pulsed on aAPCs expressing matched HLA class I allotype (**Figures 4B–E**). GFP induction was calculated by subtracting GFP-positive cells stimulated without pp65 peptide pool from GFP-positive cells stimulated with pp65 peptide pool based on analysis data of flow cytometry. In this test, a functional TCR pair was determined if the GFP induction was greater than or equal to 10%.

In the CMV pp65-specific TCR pairs restricted by HLA-A*02:01, two TCR pairs of α1-β1 (A*02:01/TCR1-1) and α3-β2 (A*02:01/TCR3-2) showed 27.1% and 23.8% of GFP induction (**Figure 4B**). In HLA-A*02:06, the one TCR pair of α2-β1 (A*02:06/TCR2-1) showed 14.2% of GFP induction (**Figure 4C**). In HLA-B*07:02, the five TCR pairs of α1-β1 (B*07:02/TCR1-1), α2-β2 (B*07:02/TCR2-2), α2-β3 (B*07:02/TCR2-3), α5-β5 (B*07:02/TCR5-5), and α6-β4 (B*07:02/TCR6-4) showed 10.8%, 26.2%, 23.6%, 22.9%, and 16.4% of GFP induction (**Figure 4D**). B*07:02/TCR1-1 showed less GFP induction than other TCR pairs of HLA-B*07:02, despite occupying the highest distribution (**Figures 3C**, **4D**). Because TCR β2 to β3 showed very similar CDR3 sequences, B*07:02/TCR2-2 and B*07:02/TCR2-3 were determined to be the same TCR, and only B*07:02/TCR2-2 was used for subsequent experiments. In HLA-B*40:06, the one TCR pair of α2-β1 (B*40:06/TCR2-1) showed 12.9% of GFP induction (**Figure 4E**).

These results demonstrate that functional TCR pairs can be easily investigated from independently obtained TCRα and TCRβ cDNAs using aAPCs and a Jurkat-based reporter cell line (J8GAB).

### Affinity of cytomegalovirus pp65-specific T-cell receptors to synthetic peptide pool or naturally processed antigen

**Figure 5:**
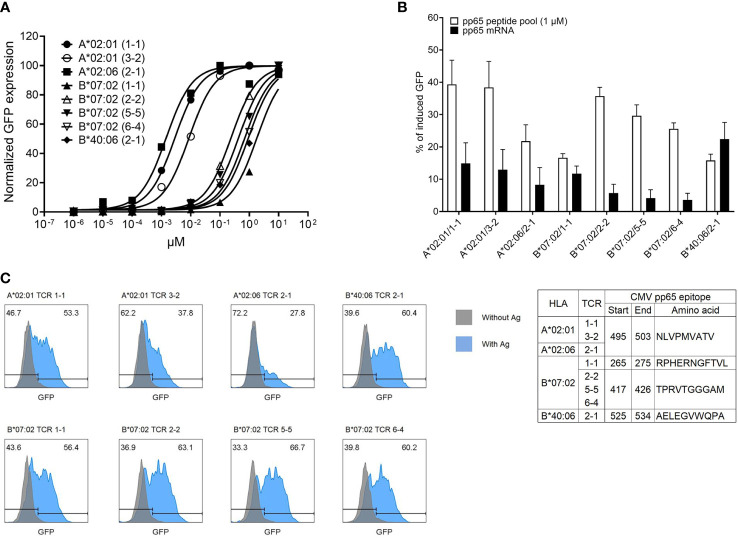
Determination of affinity and epitope of CMV pp65-specific TCR pairs. **(A)** Affinity determination of CMV pp65-specific TCR pairs *via* fold dilution of pp65 peptide pool with aAPCs expressing single HLA allotype. Each aAPC expressing HLA-A*02:01, HLA-A*02:06, HLA-B*07:02, or HLA-40:06 was pulsed with a 10-fold dilution pp65 peptide pool (from 10^1^ to 10^−6^ μM) and then were co-cultured with J8GAB cells expressing TCR pair. At 18–24 h after stimulation, the expression of induced GFP was converted to stimulus–response curve through normalization. **(B)** Comparison of responses of each TCR pair according to antigen presentation using pp65 peptide pool and pp65 mRNA transfection. Each aAPC expressing single HLA allotype was transfected IVT pp65 mRNA and co-cultured with J8GAB cells expressing TCR pair. At 18–24 h after stimulation, the expression of induced GFP by pp65 mRNA transfection was measured and compared with data by the pp65 peptide pool (1 μM). This experiment was repeated at least four times. **(C)** Determination of pp65 epitope recognized by CMV pp65-specific TCR pairs. Based on Immune Epitope Database (IEDB), we selected candidates of epitope restricted by HLA allotypes. HLA-A*02:01 and HLA-A*02:06 epitope peptide (NLVPMVATV) and HLA-B*07:02 epitope peptides (RPHERNGFTVL and TPRVTGGGAM) were pulsed on each matched aAPC expressing single HLA allotype. IVT mRNA expressing HLA-B*40:06 epitope (AELEGVWQPA) was transfected into aAPCs expressing single HLA-B*40:06 allotype. The aAPCs pulsing or expressing epitope were co-cultured with J8GAB cells expressing TCR pair. At 18–24 h after stimulation, the expression of induced GFP was measured. All selected TCR pairs reacted specifically to the presented epitope. CMV, cytomegalovirus; TCR, T-cell receptor; aAPCs, artificial antigen-presenting cells; GFP, green fluorescent protein; HLA, human leukocyte antigen.

To determine the affinity of TCR pairs, J8GAB cells expressing each TCR pair were stimulated with aAPCs pulsed with a CMV pp65 peptide pool diluted from 10^1^ to 10^−6^ μM by a 10-fold limiting dilution method. The half maximal effective concentration (EC50) of TCRs was calculated from the stimulus–response curve through normalization of % of induced GFP and was strong in the order of A*02:06/TCR2-1 (0.0014 μM), A*02:01/TCR1-1 (0.0028 μM), A*02:01/TCR3-2 (0.0082 μM), B*07:02/TCR2-2 (0.2274 μM), B*07:02/TCR5-5 (0.3945 μM), B*07:02/TCR6-4 (0.6475 μM), B*40:06/TCR2-1 (0.8763 μM), and B*07:02/TCR1-1 (1.488 μM) (**Figure 5A**). However, the order of % of induced GFP stimulated with 1 μM of peptide pool without normalization was different; A*02:01/TCR1-1 (58.6%), A*02:01/TCR3-2 (57.3%), B*07: 02/TCR2-2 (46.5%), B*07:02/TCR5-5 (41.7%), A*02:06/TCR2-1 (38%), B*07:02/TCR6-4 (29.7%), B*40:06/TCR2-1 (12.1%), and B*07:02/TCR1-1 (13.7%) were shown in the strongest order. GFP induction of A*02:06/TCR2-1 with the highest EC50 of 0.0014 μM was 40.6%, but B*07:02/TCR5-5 with a lower EC50 of 0.3945 μM was 63.7%. This shows that GFP induction and EC50 values are not necessarily correlated.

To measure the reactivity of TCR pairs to the naturally processed CMV pp65 antigen, J8GAB cells expressing each TCR pair were stimulated with aAPCs transfected with CMV pp65 mRNA. These results were compared with the GFP induction by the peptide pool (**Figure 5B**). Seven of eight TCR pairs showed stronger GFP induction upon stimulation by the peptide pool than those of naturally processed antigens. However, B*40:06/TCR2-1 with the lowest GFP induction by the peptide pool showed a higher response to the naturally processed antigen (22.4%, SEM ± 5.2) than that of the peptide pool (15.8%, SEM ± 1.9) (**Figure 5B**).

### Epitopes and alloreactivity recognized by cytomegalovirus pp65-specific T-cell receptors

To identify the epitope recognized by each TCR pair, four candidate epitopes selected based on the IEDB were used to stimulate J8GAB cells expressing each TCR pair with matched aAPCs (**Figure 5C**). A*02:01/TCR1-1, A*02:01/TCR3-2, and A*02:06/TCR2-1 recognized the same epitope of 495-503 (NLVPMVATV). Among TCR pairs restricted by HLA-B7*07:02, B*07:02/TCR2-2, B*07:02/TCR5-5, and B*07:02/TCR6-4 recognized the same epitope of 417-426 (TPRVTGGGAM), but B*07:02/TCR1-1 recognized a different epitope of 265-275 (RPHERNGFTVL). B*40:06/TCR2-1 recognized the epitope of 525-534 (AELEGVWQPA).

**Figure 6:**
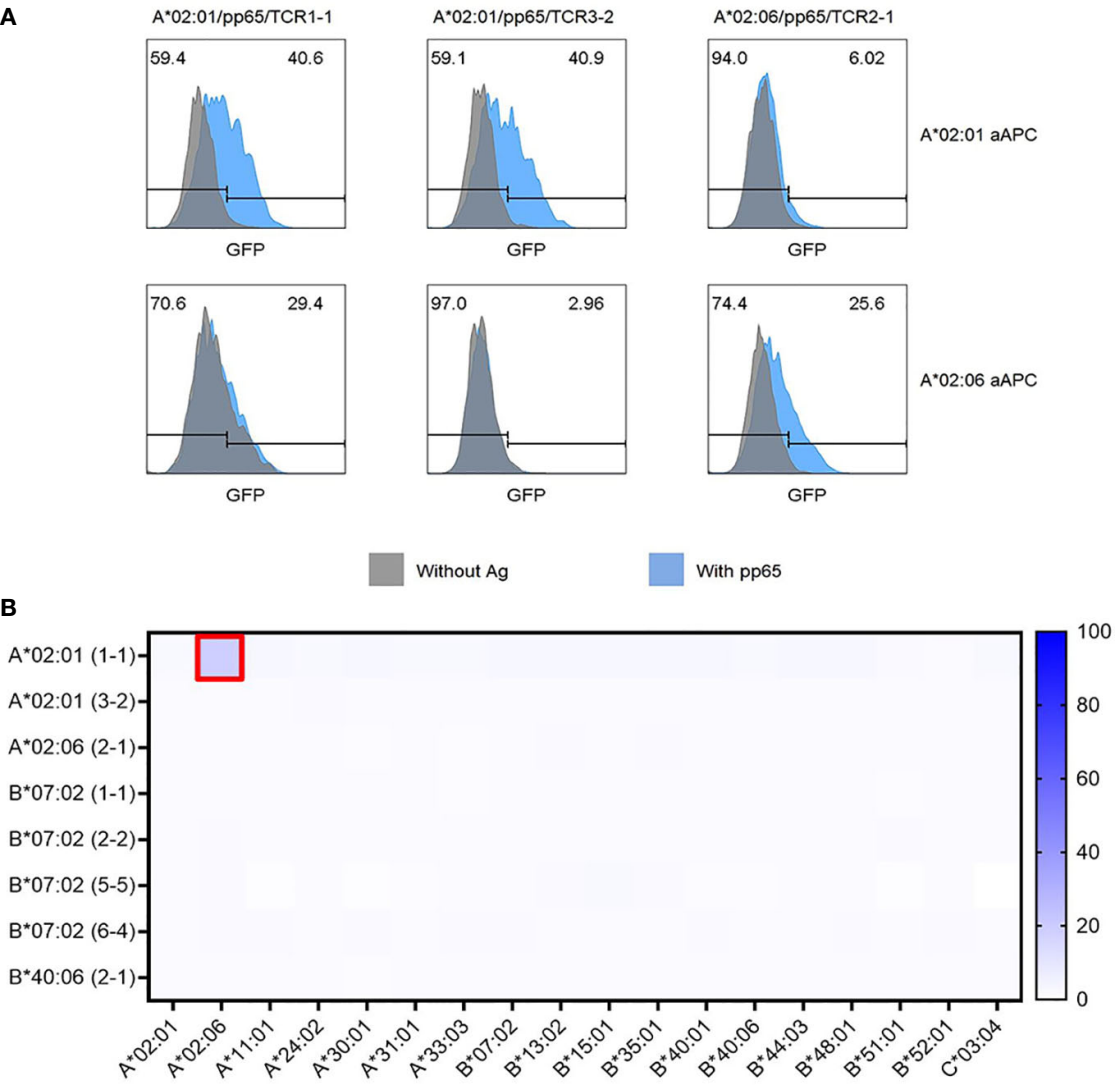
Cross-restriction and alloreactivity of CMV pp65-specific TCR pairs. **(A)** Cross-restriction analysis between three CMV pp65-specific TCR pairs recognizing the same epitope peptide presented by both HLA-A*02:01 and HLA-A*02:06. Each of the three TCR pairs (A*02:01/TCR1-1, A*02:01/TCR3-2, and A*02:06/TCR2-1) transfected J8GAB cells were co-cultured with aAPCs expressing A*02:01 or A*02:06 and expressing pp65 or not for 18 (h) It was found that they were specifically restricted to each allotype. However, A*02:01/TCR1-1 showed an alloreactivity with HLA-A*02:06 aAPCs. **(B)** Alloreactivity analysis of CMV pp65-specific TCR pairs to panel of 18 HLA class I allotypes. HLA-null aAPCs were transfected with plasmid encoding HLA class I allotype for 24 h and co-cultured with J8GAB cells transfected with each CMV pp65-specific TCR pair. A*02:01/TCR1-1 showed GFP induction against HLA-A*02:06-expressing aAPCs. TCR pairs except A*02:01/TCR1-1 did not show GFP induction with any HLA class I allotype. CMV, cytomegalovirus; TCR, T-cell receptor; aAPCs, artificial antigen-presenting cells; GFP, green fluorescent protein.

Because TCR pairs restricted by HLA-A*02:01 or HLA-A*02:06 were found to recognize the same epitope region, cross-restriction of these TCR pairs to recognize the same peptide epitope presented by different HLA allotypes was investigated. A*02:01/TCR3-2 and A*02:06/TCR2-1 specifically responded only to the peptide epitope presented by the matching HLA allotype. However, unexpectedly, A*02:01/TCR1-1 specifically recognized the peptide epitope presented by HLA-A*02:01 but showed a non-specific response to HLA-A*02:06 even in the absence of peptide (**Figure 6A**). These data showing alloreactivity rather than cross-restriction suggest that a systematic investigation of alloreactivity is needed even for TCR pairs with validated antigen specificity.

To investigate alloreactivity on 18 HLA class I allotypes, aAPC panel transfected with a single HLA class I allotype gene was co-cultured with J8GAB cells expressing each TCR pair in the absence of the pp65 antigen (**Figure 6B**). TCRs showing GFP induction of 5% or more were determined as alloreactive TCRs. Seven TCR pairs except for A*02:01/TCR1-1 showed less than 3.2% of GFP induction with 18 HLA class I allotypes (**Figure 6B**). Only A*02:01/TCR1-1 showed 15.8% of GFP induction by HLA-A*02:06 (**Figure 6B**).

## Discussion

This study was performed to establish a rapid method for the identification of functional TCRs from CMV pp65 antigen-specific T cells restricted by particular HLA allotypes in human peripheral blood. In a previous study, we analyzed T-cell responses to CMV pp65 antigen in healthy Koreans using aAPCs expressing a single HLA allotype (35). CD8-positive T-cell responses restricted by allotypes such as HLA-A*02:01, HLA-A*02:06, HLA-A*33:03, HLA-B* 07:02, HLA-B*40:06, HLA-B*51:01, and HLA-B*35:01 were observed to be relatively high. Based on these results, the CMV pp65-specific TCRs were investigated using the PBMCs derived from four donors with confirmed reactivity of CMV pp65 and restriction by a specific HLA allotype (HLA-A*02:01, HLA-A*02:06, HLA-B* 07:02, and HLA-B*40:06). A total of eight CMV pp65-specific TCRs were identified and analyzed in various aspects such as HLA restriction, affinity, epitope, and alloreactivity of each TCR. Among them, A*02:01/3-2 and B*40:06/2-1 TCRs are new CMV pp65-specific TCRs that are not reported in VDJdb. The TCRs identified in this study are restricted to a total of four HLA class I allotypes, which could cover 51.8% of the Korean population (38). It was analyzed that if TCRs restricted to the other four HLA class I allotypes (HLA-A*33:03, HLA-B*15:01, HLA-B*35:01, and HLA-B*51:01) would be added, it could cover up to 81.3%.

Isolation of antigen-specific T cells using AIMs is advantageous when the epitope peptide is unknown or a tetramer is not available. As a result of comparing the TCR repertoire of tetramer-positive sorted T cells with the TCR repertoire of T cells sorted by 4-1BB or IFN-γ capture, 4-1BB was suitable for AIM (**Figure 2**). Although all candidate TCRs had a higher distribution in the 4-1BB sorting group than in the CD8 sorting group, the A*02:01-2^nd^ Vβ sequence was dominantly higher in CD8 T cells rather than 4-1BB-positive CD8 T cells (**Figure 3A**). Since it was confirmed that the A*0201/3-2 TCR containing the A*02:01-2^nd^ Vβ sequence has functional specificity (**Figure 4B**), it is necessary to include cultured CD8 T cells as well as 4-1BB-positive CD8 T cells to analyze antigen-specific T cells after *in vitro* stimulation.

Bulk TCR sequencing by NGS does not provide information about TCR pairs, so we investigated functional TCR with a combination to find the correct pair of candidates TCRα and TCRβ sequences. We successfully established a reverse TCR cloning method capable of amplifying only specific TCR from the bulk TCR cDNA pool (**Figure 4A**). This suggests that reverse TCR cloning can be applied to overextended samples such as tumor-infiltrating lymphocytes (TILs) derived from heavily pre-treated patients. Reverse TCR cloning is the reverse of the process of conventional molecular cloning and Sanger sequencing. TCR gene presumed to be clonally expanded among the TCR repertoire sequences identified by NGS can be rapidly obtained by overlapping PCR of the variable and constant region using CDR3-specific primers. These CDR3-specific primers were designed to have a unique CDR3 sequence located at the 3′ end to increase specificity. This reverse TCR cloning method can greatly save the cost and time during TCR gene synthesis when compared to *in vitro* gene synthesis. In order to simultaneously express the TCRα and TCRβ genes at high levels, studies have been reported in which several linkers are inserted and compared (39).

In order to prevent mispairing with the endogenous TCR and to increase the expression and function of the transferred TCR, CRISPR/Cas9 system has been used (40). In this study, we measured the specificity of the TCR using a TCRαβ double knock-out cell line using the CRISPR/Cas9 system. Furthermore, in order to be more sensitive to antigen presentation by HLA class I, a Jurkat cell line expressing both the co-receptor CD8α and CD8β molecules was generated (41). Derived from the clones of the Jurkat cell line having the above characteristics, the clones expressing the most NFκB-induced GFP after stimulation with anti-CD3 and anti-CD28 antibodies were finally selected. Previously, Jurkat reporter cells for measuring T-cell response had been reported, but we wanted to establish a more sensitive reporter cell without TCR mispairing by eliminating both *TRA* and *TRB* genes and additionally expressing CD8α as well as CD8β (42, 43). In addition, the expression of GFP was in good agreement with the secretion of IL-2, which was used as a marker for TCR signaling in Jurkat cells (data not shown). This study used only the Jurkat reporter cell line for the functional analysis of TCRs. For application to TCR-T therapy, validation in primary T cells is essential, but non-specific responses may be caused by TCR mispairing. GvHD caused by TCR mispairing has been reported (44, 45). There must have been a way to overcome this through TCR modification such as murinized, cysteine-modified, and single-chain TCR (46) or TCR gene editing using the CRISPR/Cas9 system in primary T cells (40). To overcome TCR mispairing, many approaches to eliminate endogenous TCRs using RNAi or CRISPR/Cas9 systems have been studied (40, 47–50).

Four TCRs restricted to HLA-B*07:02 were identified with three TCRs specific for a pp65_417-426_ epitope (TPR) and one TCR specific for a pp65_265-275_ epitope (RPH) (**Figure 5C**). When affinity was measured using target cells pulsed with CMV pp65 peptide pool, the TCR specific to the TPR epitope showed higher affinity than the TCR specific to the RPH epitope. However, for target cells in which CMV pp65 mRNA was transferred, the TCR specific to RPH epitope showed a higher response than the TCR specific to TPR epitope. Interestingly, these results suggest that natural antigen presentation and artificial antigen presentation by peptide pulsing are different (51). Therefore, it is considered necessary to finally confirm the characteristics of TCR for clinical application by naturally processed antigen presentation.

In addition, we studied alloreactivity and cross-reactivity in the presence and absence of CMV pp65 antigen using two A*02:01 TCRs and one A*02:06 TCR that recognize pp65_495-503_ epitope (NLV) co-presented on HLA-A*02:01 and HLA-A*02:06 (**Figure 6A**). Only the A*02:01/1-1 TCR reacted to the non-self HLA allotype (**Figure 6A**). Since A*02:01/1-1 TCR reacted with A*02:06 even in the absence of antigen, it can be explained by the observed alloreactivity to HLA-A*02:06 (**Figure 6A**). Alloreactivity may be due to TCR misfocusing on non-self-MHC polymorphisms or the recognition of a repertoire of unique allo-peptides (52, 53). Alloreactivity of A*02:01/1-1 TCR appears to be due to misfocusing on non-self-MHC polymorphisms, as it is independent of the presence or absence of the pp65 antigen. This alloreactivity can cause transplant rejection and GvHD when allo-transplantation (52). Therefore, we further confirmed the alloreactivity of each TCR using our own HLA-null 293T cell line panel expressing 18 HLA class I allotypes to accurately measure the alloreactivity of the selected TCRs (**Figure 6B**).

The reverse TCR cloning we propose is a TCR cloning system that does not require single-cell-level T cells and epitope multimers. In addition, the function of TCRs can be simply confirmed without conventional molecular cloning and Sanger sequencing. A TCR identification system is simple to perform in any laboratory, provided that a Jurkat reporter cell line is present. It is believed that this simple system will help to advance TCR research more easily and quickly in the future.

## Data availability statement

The datasets presented in this study can be found in online repositories. The names of the repository/repositories and accession number(s) can be found below: https://www.ncbi.nlm.nih.gov/bioproject/PRJNA869033/, bioproject/PRJNA869033.

## Ethics statement

The studies involving human participants were reviewed and approved by Institutional Review Board of the Catholic University of Korea (MC17SESI0122). The patients/participants provided their written informed consent to participate in this study.

## Author contributions

C-HH and T-GK designed research and wrote the paper. C-HH and H-SP performed cellular and molecular experiments. C-HH and I-CB performed NGS and analyzed data. All authors contributed to the article and approved the submitted version.

## Funding

This research was supported by the BK21 program and a grant from the Korean Health Technology R&D Project, Ministry for Health and Welfare, Republic of Korea (HI14C3417).

## Acknowledgments

We thank Hyeon-Chun Park for the technical support in bioinformatics.

## Conflict of interest

The authors declare that the research was conducted in the absence of any commercial or financial relationships that could be construed as a potential conflict of interest.

## Publisher’s note

All claims expressed in this article are solely those of the authors and do not necessarily represent those of their affiliated organizations, or those of the publisher, the editors and the reviewers. Any product that may be evaluated in this article, or claim that may be made by its manufacturer, is not guaranteed or endorsed by the publisher.
