# Supplementary material for: Rapid identification of CMV-specific TCRs via reverse TCR cloning system based on bulk TCR repertoire data
Source: Front Immunol. 2022 Nov 18;13:1021067. doi: 10.3389/fimmu.2022.1021067 (PMC9716090; doi:10.3389/fimmu.2022.1021067)
Supplement: Supplementary file 1 [file Table_1.pdf]

Supplementary Material

Supplementary Tables

Supplementary Table 1. The detail information of candidates for CMV pp65-specific TCRs. List of information on V(D)J usage and CDR3 sequences of TCRs selected as candidates for CMV pp65-specific TCRs.

HLA type	Strand	Gene usages				CDR3 (Amino Acid)
		V	D	J	C	
A*02:01	α1	TRAV24		TRAJ49	TRAC	CARNTGNQFYF
	α2	TRDV1		TRAJ45	TRAC	CALGDPSGGGADGLTF
	α3	TRAV12-3		TRAJ30	TRAC	CAMSSPAIF
	β1	TRBV6-5	TRBD1	TRBJ1-2	TRBC1	CASSLSTGTAYGYTF
	β2	TRBV6-3	TRBD1	TRBJ2-1	TRBC2	CASSSAGTYNEQFF
	β3	TRBV12-4	TRBD1	TRBJ1-2	TRBC1	CASSSTAGYTF
A*02:06	α1	TRDV1		TRAJ49	TRAC	CALGDTGNQFYF
	α2	TRAV24		TRAJ21	TRAC	CASHNFNKFYF
	β1	TRBV19	TRBD2	TRBJ2-7	TRBC2	CASTAAGGAGIYEQYF
	β2	TRBV20-1	TRBD1	TRBJ2-1	TRBC2	CSARDRGVGRLGFLNEQFF
B*07:02	α1	TRAV23DV6		TRAJ48	TRAC	CAASIGNFGNEKLTF
	α2	TRAV2		TRAJ12	TRAC	CAVDRRMDSSYKLIF
	α3	TRAV10		TRAJ10	TRAC	CVVSARITGGGNKLTF
	α4	TRAV20		TRAJ22	TRAC	CAPGASGSARQLTF
	α5	TRAV10		TRAJ8	TRAC	CVVSGRLNTGFQKLVF
	α6	TRAV17		TRAJ12	TRAC	CATVERMDSSYKLIF
	β1	TRBV4-3		TRBJ1-1	TRBC1	CASSPQRNTEAFF
	β2	TRBV7-9	TRBD2	TRBJ2-2	TRBC2	CASSLREGANTGELFF
	β3	TRBV7-9	TRBD1	TRBJ2-2	TRBC2	CASSKRQGANTGELFF
	β4	TRBV7-9	TRBD1	TRBJ2-1	TRBC2	CASSLKGVSSYNEQFF
	β5	TRBV27	TRBD1	TRBJ1-2	TRBC1	CASRLGAGGGNYGYTF
	β6	TRBV20-1	TRBD1	TRBJ1-2	TRBC1	CTEPDSPTSSLNYGYTF
B*40:06	α1	TRAV22		TRAJ26	TRAC	WRSE_FVF
	α2	TRAV14DV4		TRAJ3	TRAC	CAMREGLLYSSASKIIF
	α3	TRAV14DV4		TRAJ10	TRAC	CAMRDLGGLTGGGNKLTF
	α4	TRAV23DV6		TRAJ16	TRAC	CAATTTDGQKLLF
	β1	TRBV4-3	TRBD2	TRBJ2-4	TRBC2	CASSQESSGRATAKNIQYF
	β2	TRBV4-3	TRBD1	TRBJ2-4	TRBC2	CASSQEGQGRSGPKNIQYF
	β3	TRBV20-1	TRBD2	TRBJ2-7	TRBC2	CSAPNGPGGLEQYF
