# Supplementary material for: Rapid identification of CMV-specific TCRs via reverse TCR cloning system based on bulk TCR repertoire data
Source: Front Immunol. 2022 Nov 18;13:1021067. doi: 10.3389/fimmu.2022.1021067 (PMC9716090; doi:10.3389/fimmu.2022.1021067)
Supplement: Supplementary file 2 [file Table_2.pdf]

Supplementary Material

Supplementary Tables

Supplementary Table 2. The details of primers for reverse TCR cloning. List of primers and each nucleotide sequences using to synthesize IVT templates for reverse TCR cloning.

Primer name	Purpose	Sequence (5'→3')
Universal switch_F	Used to amplify templates for CDR3 specific PCR	AAGCAGTGGTATCAACGCAGAGTACGGG
TRAC_R		TCAGCTGGACCACAGCCGCA
TRBC1_R		TCAGAAATCCTTTCTCTTGA
TRBC2_R		CTAGCCTCTGGAATCCTTTC
T7_TRDV1_F	Used for CDR3 specific PCR	CCCAAGCTTGCCACCATGCTGTTCTCCAGCCTGCTGTGTGT
T7_TRAV1-1_F		CCCAAGCTTGCCACCATGTGGGAGCTTTCCTTCTCTATG
T7_TRAV1-2_F		CCCAAGCTTGCCACCATGTGGGGAGTTTTCCTTCTTTAT
T7_TRAV2_F		CCCAAGCTTGCCACCATGGCTTTGCAGAGCACTCTGGGGG
T7_TRAV3_F		CCCAAGCTTGCCACCATGGCCTCTGCACCCATCTCGATG
T7_TRAV4_F		CCCAAGCTTGCCACCATGAGGCAAGTGGCGAGAGTGATCGT
T7_TRAV9-2_F		CCCAAGCTTGCCACCATGAACTATTCTCCAGGCTTAGTATC
T7_TRAV10_F		CCCAAGCTTGCCACCATGAAAAAGCATCTGACGACCTTCT
T7_TRAV12-3_F		CCCAAGCTTGCCACCATGATGAAATCCTTGAGAGTTTTACT
T7_TRAV13-1_F		CCCAAGCTTGCCACCATGACATCCATTCGAGCTGTATTTA
T7_TRAV14DV4_F		CCCAAGCTTGCCACCATGTCACTTTCTAGCCTGCTGAAGGT
T7_TRAV17_F		CCCAAGCTTGCCACCATGGAAACTCTCCTGGGAGTGTCTTT
T7_TRAV20_F		CCCAAGCTTGCCACCATGGAGAAAATGTTGGAGTGTGCATT
T7_TRAV21_F		CCCAAGCTTGCCACCATGGAGACCCTCTTGGGCCTGCTTAT
T7_TRAV22_F		CCCAAGCTTGCCACCATGAAGAGGATATTGGGAGCTCTGC
T7_TRAV23DV6_F		CCCAAGCTTGCCACCATGGACAAGATCTTAGGAGCATCAT
T7_TRAV24_F		CCCAAGCTTGCCACCATGGAGAAGAATCCTTTGGCAGCCCC
T7_TRAV29DV5_F		CCCAAGCTTGCCACCATGGCCATGCTCCTGGGGGCATCAGT
T7_TRAV38-2DV8_F		CCCAAGCTTGCCACCATGGCATGCCCTGGCTTCCTGTGGGC
T7_TRAV41_F		CCCAAGCTTGCCACCATGGTGAAGATCCGGCAATTTTTGTT
T7_TRBV2_F		CCCAAGCTTGCCACCATGGATACCTGGCTCGTATGCTGGGC
T7_TRBV4-1, 2, 3_F		CCCAAGCTTGCCACCATGGGCTGCAGGCTGCTCTGCTGTGC
T7_TRBV6-3_F		CCCAAGCTTGCCACCATGAGCCTCGGGCTCCTGTGCTGTGG
T7_TRBV6-5_F		CCCAAGCTTGCCACCATGAGCATCGGCCTCCTGTGCTGTGC
T7_TRBV7-8_F		CCCAAGCTTGCCACCATGGGCACCAGGCTCCTCTGCTGGGT
T7_TRBV7-9_F		CCCAAGCTTGCCACCATGGGCACCAGCCTCCTCTGCTGGAT
T7_TRBV12-4_F		CCCAAGCTTGCCACCATGGGCTCCTGGACCCTCTGCTGTGT
T7_TRBV16_F		CCCAAGCTTGCCACCATGAGCCCAATATTCACCTGCATCAC
T7_TRBV19_F		CCCAAGCTTGCCACCATGAGCAACCAGGTGCTCTGCTGTGT
T7_TRBV20-1_F		CCCAAGCTTGCCACCATGCTGCTGCTTCTGCTGCTTCTGG
T7_TRBV27_F		CCCAAGCTTGCCACCATGGGCCCCCAGCTCCTTGGCTATGT
T7_TRBV28_F		CCCAAGCTTGCCACCATGGGAATCAGGCTCCTCTGTCGTG
A0201_a1_CDR3_F		TGTGCCCGGAACACCGGTAA
A0201_a1_CDR3_R		AAAATAGAACTGGTTACCGG
A0201_a2_CDR3_F		TGTGCTCTTGGTGATCCTTC
A0201_a2_CDR3_R		AAAGGTGAGTCCGTCAGCAC
A0201_a3_CDR3_F		TGTGCAATGAGCTCCCCAGC
A0201_a3_CDR3_R		AAAGATCGCTGGGGAGCTCA
A0201_b1_CDR3_F		TGTGCCAGCAGTTTATCGAC
A0201_b1_CDR3_R		GAAGGTGTAGCCATAGGCTG
A0201_b2_CDR3_F		TGTGCCAGCAGTTCCGCCGG
A0201_b2_CDR3_R		GAAGAACTGCTCATTGTAGG
A0201_b3_CDR3_F		TGTGCCAGCAGTTCGACAGC
A0201_b3_CDR3_R		GAAGGTGTAGCCAGCTGTCG

A0206_a1_CDR3_F	Used for CDR3 specific PCR	TGTGCTCTTGGGGACACCGG
A0206_a1_CDR3_R		AAAATAGAACTGGTTACCGG
A0206_a2_CDR3_F		TGTGCCTCCCACAACTTCAA
A0206_a2_CDR3_R		AAAGTAAAATTTGTTGAAGT
A0206_b1_CDR3_F		TGTGCCAGTACCGCCGCGGG
A0206_b1_CDR3_R		GAAGTACTGCTCGTAGATCC
A0206_b2_CDR3_F		TGCAGTGCTAGAGATCGGGG
A0206_b2_CDR3_R		GAAGAACTGCTCATTTAGAA
B0702_a1_CDR3_F		TGTGCAGCAAGCATAGGAAA
B0702_a1_CDR3_R		AAAGGTTAATTTCTCATTTC
B0702_a2_CDR3_F		TGTGCTGTGGACCGCCGGAT
B0702_a2_CDR3_R		GAAGATCAATTTATAGCTGC
B0702_a3_CDR3_F		TGTGTGGTGAGCGCGAGGAT
B0702_a3_CDR3_R		AAAGGTGAGTTTGTTTCCTC
B0702_a4_CDR3_F		TGTGCTCCTGGGGCTTCTGG
B0702_a4_CDR3_R		AAAGGTCAGTTGCCTTGCAG
B0702_a5_CDR3_F		TGTGTGGTGAGCGGGAGGCT
B0702_a5_CDR3_R		AAATACAAGTTTCTGAAAGC
B0702_a6_CDR3_F		TGTGCTACGGTCGAAAGGAT
B0702_a6_CDR3_R		GAAGATCAATTTATAGCTGC
B0702_b1_CDR3_F		TGCGCCAGCAGCCCTCAGAG
B0702_b1_CDR3_R		AAAGAAAGCTTCAGTGTTTC
B0702_b2_CDR3_F		TGTGCCAGCAGCCTAAGAGA
B0702_b2_CDR3_R		AAAAAACAGCTCCCCGGTGT
B0702_b3_CDR3_F		TGTGCCAGCAGCAAACGACA
B0702_b3_CDR3_R		AAAAAACAGCTCCCCGGTGT
B0702_b4_CDR3_F		TGTGCCAGCAGCTTAAAGGG
B0702_b4_CDR3_R		GAAGAACTGCTCATTGTAGG
B0702_b5_CDR3_F		TGTGCCAGCAGATTGGGAGC
B0702_b5_CDR3_R		GAAGGTGTAGCCATAGTTAC
B0702_b6_CDR3_F		TGCACCGAACCCGACAGCCC
B0702_b6_CDR3_R		GAAGGTGTAGCCATAGTTTA
B4006_a1_CDR3_F		TGGCGGTCAGAATTTTGTCT
B4006_a1_CDR3_R		AAAGACAAAATTCTGACCGC
B4006_a2_CDR3_F		TGTGCAATGAGAGAGGGATT
B4006_a2_CDR3_R		AAAGATTATCTTGGAAGCAC
B4006_a3_CDR3_F		TGTGCAATGAGAGACCTCGG
B4006_a3_CDR3_R		AAAGGTGAGTTTGTTTCCTC
B4006_a4_CDR3_F		TGTGCAGCAACCACCACAGA
B4006_a4_CDR3_R		AAAGAGCAGCTTCTGGCCAT
B4006_b1_CDR3_F		TGCGCCAGCAGCCAAGAGTC
B4006_b1_CDR3_R		GAAGTACTGAATGTTTTTGG
B4006_b2_CDR3_F		TGCGCCAGCAGCCAAGAAGG
B4006_b2_CDR3_R		GAAGTACTGAATGTTTTTGG
B4006_b3_CDR3_F		TGCAGTGCTCCTAACGGTCC
B4006_b3_CDR3_R		GAAGTACTGCTCGAGCCCAC
TRAC_pA_R		AGTCAGATGCTCAAGTCAGCTGGACCACAGCCGCA
TRBC1_pA_R		AGTCAGATGCTCAAGTCAGAAATCCTTTCTCTTGA
TRBC2_pA_R		AGTCAGATGCTCAAGCTAGCCTCTGGAATCCTTTC
CMV pp65_T7_F	Used to amplify CMV pp65 templates for IVT	CCCAAGCTTGCCACCATGGAATTCATGGAGTCGCG
T2A_R		AGGGCCGGGATTCTCCTCCACGTCACCGCATGTTAGAAGACTTCC TCTGCCCTC
T2A_TagBFP_F		GAGAATCCCGGCCCTATGTCCGAGCTGATTAAGGA
TagBFP_pA_R		AGTCAGATGCTCAAGTCAATTAAGCTTGTGCCCCA
T7_B4006 epitope_F		AAGCTTGCCACCATGGAATTCGCCGAATTGGAAGGCGTATGGCA GCCCGCTGAGGGCAGAGGAAGTCTTCT
T7_plus_F	Used in overlapping PCR to synthesize the final IVT templates	AAATTAATACGACTCACTATAGGGAGACCCAAGCTTGCCACCATG
Beta-globin pA_R		ACAAAAAATTCCAACACACTATTGCAATGAAAATAAATTTCCTTT ATTAGCCAGAAGTCAGATGCTCAAG
