# Supplementary material for: Rapid identification of CMV-specific TCRs via reverse TCR cloning system based on bulk TCR repertoire data
Source: Front Immunol. 2022 Nov 18;13:1021067. doi: 10.3389/fimmu.2022.1021067 (PMC9716090; doi:10.3389/fimmu.2022.1021067)
Supplement: Supplementary file 3 [file Image_1.pdf]

Supplementary Material

1 Supplementary Figure

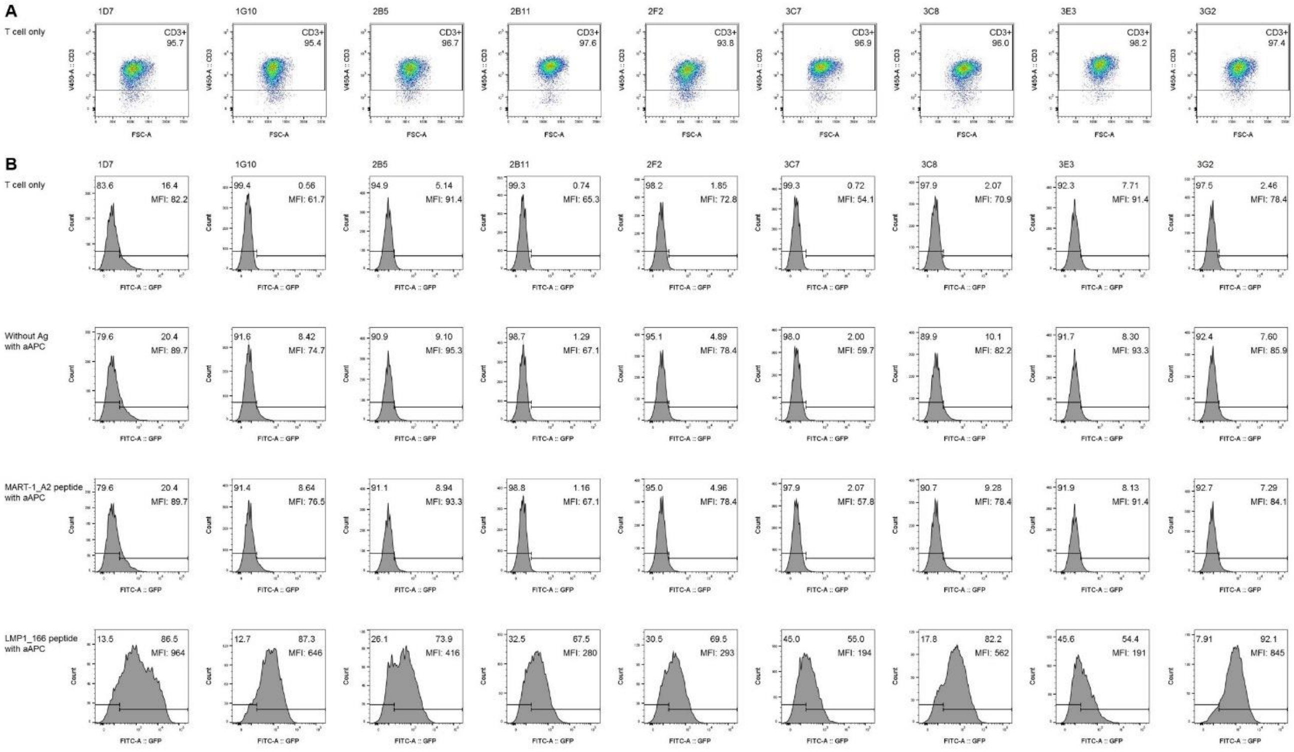

Supplementary Figure S1. Comparison of antigen-specific responses in TCRαβ double knock-out Jurkat reporter clones. (A) CD3 expression of TCRαβ double knock-out Jurkat reporter clones after LMP1-specific TCR mRNA transfection. **(B)** Measurement of GFP induced in TCRαβ double knock-out Jurkat reporter clones after stimulation with aAPCs. MART-1 A2 peptide used as irrelevant peptide control.
