# Supplementary material for: Rapid identification of CMV-specific TCRs via reverse TCR cloning system based on bulk TCR repertoire data
Source: Front Immunol. 2022 Nov 18;13:1021067. doi: 10.3389/fimmu.2022.1021067 (PMC9716090; doi:10.3389/fimmu.2022.1021067)
Supplement: Supplementary file 4 [file Image_2.pdf]

Supplementary Material

1 Supplementary Figure

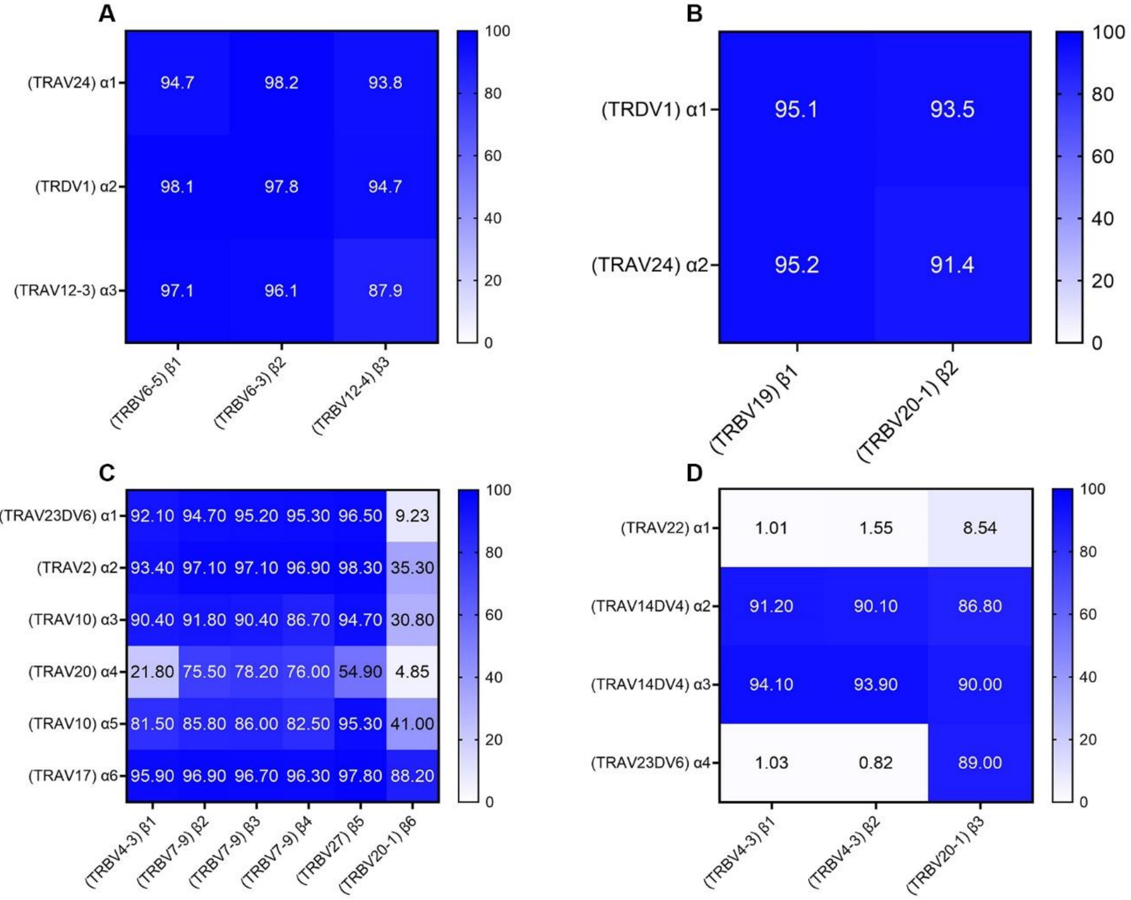

Supplementary Figure S2. CD3 expression profiles of candidate TCRα and TCRβ mRNA combinations. (A) Heatmap results for CD3 expression after transfection of candidate TCRα and TCRβ mRNA combinations of HLA-A*02:01. **(B)** Heatmap results for CD3 expression after transfection of candidate TCRα and TCRβ mRNA combinations of HLA-A*02:06. **(C)** Heatmap results for CD3 expression after transfection of candidate TCRα and TCRβ mRNA combinations of HLA-B*07:02. **(D)** Heatmap results for CD3 expression after transfection of candidate TCRα and TCRβ mRNA combinations of HLA-B*40:06.
